# Genotyping-by-Sequencing Facilitates a High-Density Consensus Linkage Map for *Aegilops umbellulata*, a Wild Relative of Cultivated Wheat

**DOI:** 10.1534/g3.117.039966

**Published:** 2017-03-29

**Authors:** Erena A. Edae, Pablo D. Olivera, Yue Jin, Matthew N. Rouse

**Affiliations:** * United States Department of Agriculture-Agricultural Research Service (USDA-ARS), Cereal Disease Laboratory, St. Paul, Minnesota 55108; † Department of Plant Pathology, University of Minnesota, St. Paul, Minnesota 55108

**Keywords:** consensus map, GBS, high-density linkage map, *Aegilops umbellulata*, wild relatives

## Abstract

High-density genetic maps are useful to precisely localize QTL or genes that might be used to improve traits of nutritional and/or economical importance in crops. However, high-density genetic maps are lacking for most wild relatives of crop species, including wheat. *Aegilops umbellulata* is a wild relative of wheat known for its potential as a source of biotic and abiotic stress resistance genes. In this work, we have developed a framework consensus genetic map using two biparental populations derived from accessions PI 298905, PI 542369, PI 5422375, and PI 554395. The framework map comprised 3009 genotype-by-sequence SNPs with a total map size of 948.72 cM. On average, there were three SNPs per centimorgan for each chromosome. Chromosome 1U was the shortest (66.5 cM), with only 81 SNPs, whereas the remaining chromosomes had between 391 and 591 SNP markers. A total of 2395 unmapped SNPs were added to the linkage maps through a recombination frequency approach, and increased the number of SNPs placed on the consensus map to a total of 5404 markers. Segregation distortion was disproportionally high for chromosome 1U for both populations used to construct component linkage maps, and thus segregation distortion could be one of the probable reasons for the exceptionally reduced linkage size for chromosome 1U. From comparative analysis, *Ae*. *umbellulata* chromosomes except 4U showed moderate to strong collinearity with corresponding homeologous chromosomes of hexaploid wheat and barley. The present consensus map may serve as a reference map in QTL mapping and validation projects, and also in genome assembly to develop a reference genome sequence for *Ae. umbellulata*.

Wild relatives of many crop species have been used as sources of useful genes in crop improvement programs (Hajjar and Hodgkin 2007). Several genes derived from wild relatives have already been deployed in cultivated wheat varieties over the past decades, and have played a significant role in wheat improvement worldwide (Prescott-Allen and Prescott-Allen 1988; Wulff and Moscou 2014). Species of the genus *Aegilops* have been used successfully in wheat wide-crossing programs (Zaharieva and Monneveux 2006; Gong *et al.* 2014), and in the production of several substitution, addition, and translocation lines containing chromosome introgressions from diploid *Aegilops* species such as *Ae. umbellulata*, *Ae. comosa*, *Ae. speltoides*, and *Ae. markgrafii* (Jiang *et al.* 1994; Friebe *et al.* 1996; Schneider *et al.* 2008; Kilian *et al.* 2011). *Aegilops* species are annual grasses, and most of them are considered as self-fertile, with an outcrossing rate similar to that of wheat (Zaharieva and Monneveux 2006). *Aegilops umbellulata* (2*n* = 2*x* = 14, UU genome), a Mediterranean and western Asiatic grass, is one of the 11 diploid species in the *Aegilops* genera (Van Slageren 1994) that possesses seven pairs of chromosomes. *Ae. umbellulata* has been identified as a source of resistance to stem rust (caused by *Puccinia graminis* f. sp. *tritici*) (Ozgen *et al.* 2004), powdery mildew (caused by *Blumeria graminis* f. sp. *tritici*), Hessian fly (*Mayetiola destuctor*), and greenbug (*Schizaphis graminum*) (Gill *et al.* 1985). It was the species from which leaf rust resistance gene *Lr9* was transferred to cultivated hexaploid wheat (Sears 1956). Hybridization barriers such as reduced embryo development and stunted growth, or sterility of F_1_ hybrids between *Ae. umbellulata* and *Triticum* sp. have been overcome through techniques such as embryo rescue and bridge crossing (Sears 1956; Hadzhiivanova *et al.* 2012). Consequently, *Triticum aestivum–Ae. umbellulata* addition lines (Kimber *et al.* 1967; Friebe *et al.* 1996), substitution lines (Reader and Miller 1987), and translocation lines (Koebner and Shepherd 1987) have been produced. However, as with all wild relatives of cultivated crops, genomic resources for *Ae. umbellulata* have been limited, and, until now, suitable molecular markers have not been available for this species (Molnar *et al.* 2013). Specifically, *Ae. umbellulata* is lacking genomic resources such as genetic linkage maps, genome-specific SNP markers, and a reference sequence.

Genetic linkage maps have wide applications in genetics and genomics research. Availability of a genetic linkage map is a prerequisite for QTL mapping (Lander and Bosttein 1989; Doerge 2002) and map-based gene cloning (Huang *et al.* 2003; Saintenac *et al.* 2013). The availability of linkage maps also allows efficient utilization of the genetic diversity in wild relatives of cultivated crops (Tanksley and McCouch 1997). Consensus linkage maps are useful to compare locations of QTL identified in independent populations (Westbrook *et al.* 2015). There is a wide gap between domesticated crop species and their wild relatives in terms of available genetic resources. Multiple genetic linkage maps have already been developed for cereal crop species, and consensus genetic maps have been constructed for hexaploid wheat (Somers *et al.* 2004; Wang *et al.* 2014), durum wheat (Maccaferri *et al.* 2014, 2015), barley (Li *et al.* 2010), and rye (Milczarski *et al.* 2011).

Recent advances in high-throughput DNA sequencing technologies have enabled the discovery of a large number of SNPs in major crops such as wheat (Poland *et al.* 2012), rice (McCough *et al.* 2016), and maize (Lu *et al.* 2015). Genotype-by-sequencing (GBS), a complexity-reduction sequencing method for large genomes (Elshire *et al.* 2011), is becoming commonplace in *de novo* genetic map construction in many species. GBS typically provides a large number of SNP markers per mapping population. High resolution genetic maps that can be created with a large numbers of SNP markers are useful not only to precisely map the position of QTL/genes but also to order scaffolds in a genome assembly as done for hexaploid wheat (IWGSC 2014) and barley (Mayer *et al.* 2012; Mascher *et al.* 2013) for developing high-quality reference genome sequences. Although genome reference sequences are available for many major crops species, orphan crops and wild relatives of the cultivated crops are still lacking ordered reference genome sequences. Here, we report a GBS-based high-density consensus linkage map constructed for *Ae. umbellulata*.

## Materials and Methods

### Mapping populations

Two F_2_ *Ae. umbellulata* populations were used for linkage map construction. The first population (hereafter *Aeupop1*) consisted of a total of 140 F_2_ individuals that were derived from a cross made between two accessions of *Ae. umbellulata*: PI 298905, originally collected from Manisa, Turkey, and resistant to wheat stem rust pathogen *P. graminis* f. sp. *tritici* race TTTTF (isolate 01MN84A-1-2), and PI 542369, originally collected from Usak, Turkey, and susceptible to race TTTTF. Similarly, the second population (hereafter *Aeupop2*) was derived from a cross made between accessions PI 542375 (originally collected from Denizli, Turkey, and resistant to race TTTTF) and PI 554395 (originally collected from Diyarbakir, Turkey, and susceptible to race TTTTF). There were a total of 154 F_2_ individuals derived from this cross.

### GBS library preparation and sequencing

Leaf tissue was collected from each F_2_ individual and the four parents at the seedling stage, and DNA was extracted for each individual following the BioSprint protocol (https://www.qiagen.com/us/shop/sample-technologies/dna/genomic-dna/biosprint-96-dna-plant-kit/). A total of two GBS libraries was constructed from a single pool of samples per population [a pool of 142 samples for *Aeupop1*, and a pool of 156 samples for *Aeupop2* following a GBS protocol with the two restriction enzymes *Pst*I (CTGCAG) and *Msp*I (CCGG) (Poland *et al.* 2012)]. Two bar-coded adaptors were used for each sample. The four parents were sequenced to a depth of 6× compared to that of the F_2_ individuals. The library was sequenced on the Illumina HiSequation 2000 platform.

### GBS SNP calling

Raw sequence data of the two populations were processed together for SNPs discovery with the UNEAK algorithm (Lu *et al.* 2013) implemented in TASSEL 3.0 (Bradbury *et al.* 2007). The UNEAK parameters were set as follows: maximum number of expected reads per sequence file at 3,000,000,000; restriction enzymes used for library construction were Pst*I*–*Msp*I; minimum number of tags required for output was 16; maximum tag number in the merged tag counts was 200,000,000; option to merge multiple sample per line was “yes;” error tolerance rate at 0.01; minimum/maximum minor allele frequencies (MAF) were 0.01 and 0.5; and minimum/maximum call rates were 0 and 1. SNPs with up to 80% missing data points were retained for subsequent data analysis. The discovered SNP data were managed separately for each population for downstream analysis. SNP alleles of the two parents were used to further filter the data where SNPs were required to be homozygous, polymorphic (opposite allele calls in parents), and no missing data points for the two parents of each population. Only SNPs that passed these filtering criteria were converted into parental genotypes.

### Linkage map construction

SNP data were first converted into parental genotypes for polymorphic SNPs with no missing or heterozygous genotypes for both parents. SNPs with minor allele frequency (MAF) <20%, percent heterozygosity >80%, and proportion of missing data points >10% were also removed. SNPs with distorted segregation at p <0.01 were also removed. A total of 1841 and 1403 SNPs for *Aeupop1* and *Aeupop2* passed these filtering criteria, respectively. These quality-filtered SNP markers were retained for framework linkage map construction for each population and consensus map construction. Linkage map construction was done with MSTMap algorithm (Wu *et al.* 2008) with the following parameters: Distance function Kosambi, cut_off_p_value set at 10^−8^, no_map_dist set at 15, no_map_size set at 0, missing_threshold set at 0.10, estimate_before_clustering set at “no,” detect_bad_data set at “yes,” and objective function set at “ML” for both populations, except that cut_off_p_value was set at 10^−9^ for *Aeupop1*. An inflated linkage map size from the first round of mapping was stabilized using a sliding window approach (Huang *et al.* 2009) to correct genotypic error. The genotypic data were processed, with modification, with Python written scripts that were implemented in Genotype-Corrector software (Miao, C., J. Fang, P. Liang, X. Zhang, and H. Tang, unpublished data). Linkage maps were also constructed using AntMap software (Iwata and Ninomiya 2006) using the corrected data sets to confirm the consistency of marker order and genetic positions.

### GBS SNP annotation With gene models and transcripts

For the purpose of determining the number of genic GBS SNPs, sequence similarity searches were completed for all 41,933 SNP tags of *Ae. umbellulata* in the local databases created for gene models of hexaploid wheat and barley, and cDNA assemblies of *Ae. tauschii*, *Triticum urartu*, and *Ae. umbellulata*. For all species, maximum *e*-value of 10^−10^ and percent of identity >80% were used as thresholds to define the best hit for each GBS SNP tag. Only one best hit per SNP tag was retained. The retrieved best hits of each species were merged with the SNPs on the consensus map.

### Consensus linkage map construction

Composite maps from the two populations were constructed for each chromosome using R package LPmerge (Endelman and Plomion 2014), which uses marker names and genetic distances as input instead of recombination frequencies. With this approach, the component linkage maps are integrated into one map by minimizing the mean absolute error between composite map and component maps using linear programming. A maximum interval size (*K*) between bins ranging from *K* = 1 to *K* = 4 were tested, and the consensus map at each *K* value was evaluated based on the minimum root mean-squared error (RMSE) and SD between the consensus map and the component linkage maps. Population sizes (139 and 149 after removing individuals with <45% missing marker data) were used to weigh in the objective function. A consensus map at K with minimum mean RMSE and SD was selected for each chromosome. Two consensus maps were constructed: a consensus map constructed using SNP markers with known genetic map position (*hereafter framework consensus map*), and a consensus map consisting of both markers with known genetic map position and SNP markers placed on the linkage maps using minimum recombination frequency values (hereafter high-density map). For the construction of the high-density consensus map, initially, recombination frequencies were calculated among markers that passed filtering criteria ≤40% missing data points, polymorphic between the parents, and no missing data for both parents. The calculation of recombination frequencies was done separately for each mapping population. For each unmapped marker (SNP with unknown genetic map position), recombination frequency values corresponding to all other mapped markers (SNPs with known genetic map position) were extracted and sorted from minimum to maximum recombination frequency values. Only recombination frequency values ≤0.1 were used to assign the unmapped markers to the closest markers on a linkage map. Each unmapped marker was assigned to the genetic map position of the marker with which it had the smallest recombination frequency value. In cases where two mapped markers had a tie in minimum recombination frequency, the unmapped marker was placed between the two mapped markers using their average genetic distance.

### Assessing the syntenic relationship between Ae. umbellulata and both hexaploid wheat and barley

Chromosome assignment of the linkage groups of the framework map was accomplished utilizing the draft genome assembly of hexaploid wheat v0.4 and the barley genome assembly (ftp://ftpmips.helmholtz-muenchen.de/plants/barley/public_data/). A total of 41,933 *Ae. umbellulata* SNP tags were similarity-searched using “blastn” in the locally created database for both species assemblies. Only best hits significant at e value of 10^–5^ were retrieved for subsequent analysis. Linkage groups were assigned to respective chromosomes of Ae. umbellulata comparing the best hits found for each wheat chromosome. The degree of collinearity of Ae. umbellulata with hexaploid wheat and barley was determined by calculating Spearman’s rank correlation coefficients (*ρ*) based on the following formula:

*ρ* = 1− 6∑*d_i_*^2^/[*n*(*n*^2^−1)], where *ρ* is Spearman’s rank correlation, ∑*d_i_*^2^ is the summation of rank differences, and *n* is the number pairs to be ranked.

### Segregation distortion analysis

Significant deviation from the expected Mendelian genotypic frequencies (1:2:1 genotypic ratio for F_2_ population) was tested using chi-square tests (*α* ≤0.01). We used SNP markers added to the framework map with a recombination frequency approach. The patterns of segregation distortion across chromosome regions were assessed by plotting negative logarithms of p-values [−log_10_ (p-values)] *vs.* consensus genetic distance (centimorgan). The proportion of distorted markers was also calculated for each chromosome. The frequencies of the three genotypic classes were plotted against genetic distance to assess the direction of segregation distortion. Distorted regions were also identified for each chromosome. Distorted regions (DR) were defined as regions of a chromosome with two or more significantly distorted markers (*α* ≤0.01).

### Data availability

All necessary data we used to draw conclusions in this article are represented either within the article or within Supplemental Material. Supplemental Material, Table S1 consists of linkage maps constructed for both populations using MSTMap and AntMap softwares. Table S2 contains the high density consensus map, framework map, and physical positions of SNPs in Chinese Spring wheat assembly, and rank correlation coefficient data are also available in Table S3.

## Results and Discussion

### Component maps and framework map construction

In this study we report a consensus linkage map constructed based on individual maps of two F_2_ populations of *Ae. umbellulata* using GBS platform. First, the component maps of the two mapping populations were constructed with MSTMap software using quality-filtered genotypic datasets except that the weakly distorted SNPs (*α* >0.01) were kept in the datasets as segregation distortion is a common phenomenon in many crop species such as maize (Sharopova *et al.* 2002), sorghum (Mace *et al.* 2009), potato (Van Os *et al.* 2006), rye (Senft and Wricke 1996; Korzun *et al.* 2001), and triticale (Alheit *et al.* 2011). By removing SNP markers that were highly distorted (*α* <0.01), we successfully constructed component linkage maps using two populations for all chromosomes.

**Figure 1:**
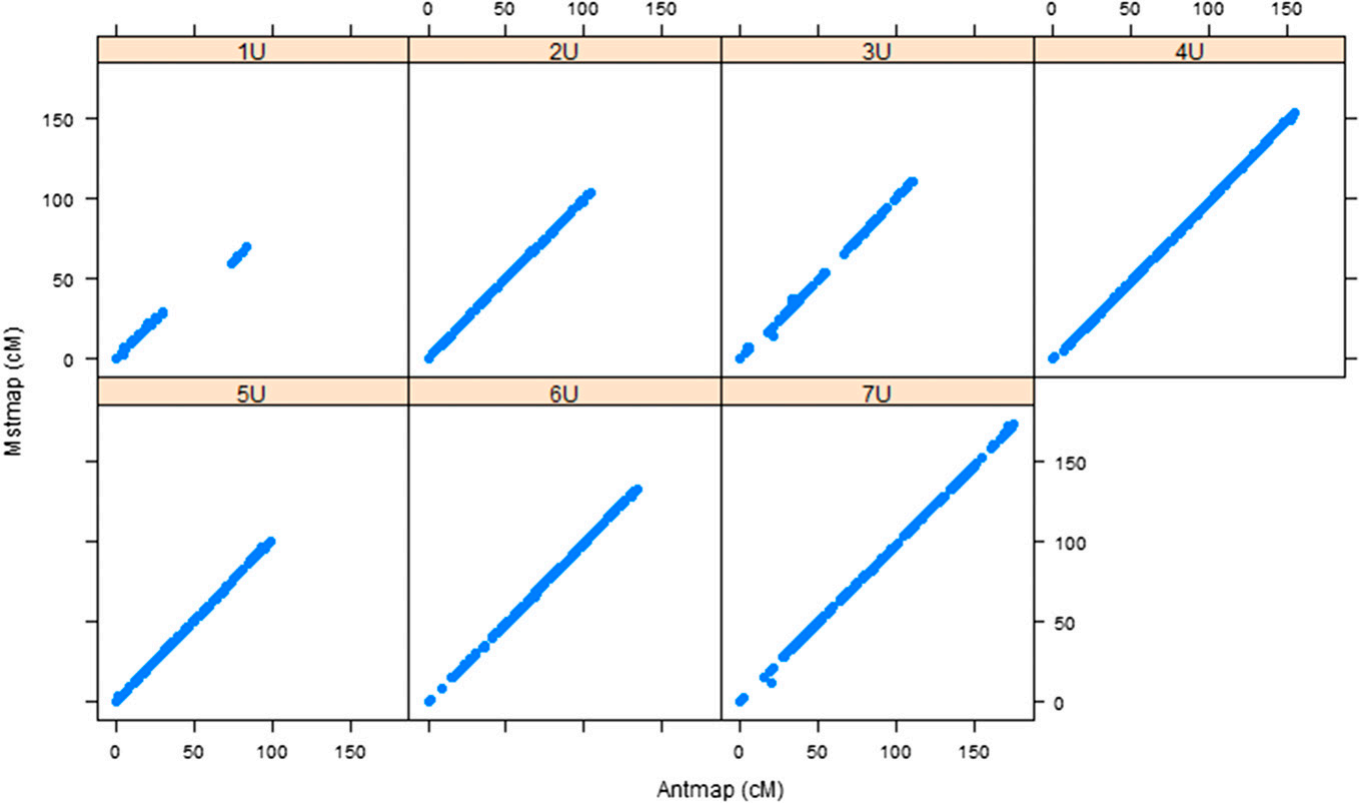
Comparison of linkage maps constructed with MSTMap and Antmap algorithms for *Aeupop1*. The *x-axis* indicates the genetic distance centimeter based on Antmap software, whereas the *y-axis* indicates the genetic distance centimeter based on MSTMap software for each chromosome.

**Figure 2:**
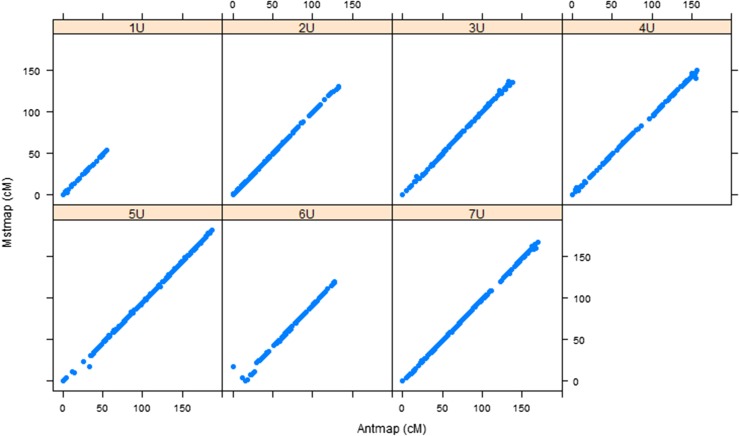
Comparison of linkage maps constructed with MSTMap and Antmap algorithms for *Aeupop2*. The *x-axis* indicates the genetic distance (centimorgan) based on Antmap software, whereas the *y-axis* indicates the genetic distance (centimorgan) based on MSTMap software for each chromosome.

For the purpose of assessing the accuracy of the individual maps, we confirmed the consistency of marker order and genetic positions through remapping with an independent mapping algorithm implemented in AntMap software, and found strong agreement between corresponding linkage groups for both populations (Figure 1, Figure 2, and Table S1). Then a framework consensus map comprised of 3009 GBS markers of two populations was constructed (*Aeupop1* and *Aeupop2*) (Table 1).

**Table 1:** Distribution of markers and lengths of the framework consensus genetic map constructed for *Ae. umbellulata* using two biparental populations

Chromosomes	No. of Markers	Map Size (cM)	Average Gap Size (cM)	SNP/cM
1U	81	66.47	0.83	1.2
2U	446	131.19	0.30	3.4
3U	391	110.64	0.28	3.5
4U	523	149.32	0.29	3.5
5U	451	181.09	0.40	2.5
6U	526	133.25	0.25	4.0
7U	591	176.82	0.30	3.3
Total	3009	948.72		
Mean	429.9	135.53		3.1

**Figure 3:**
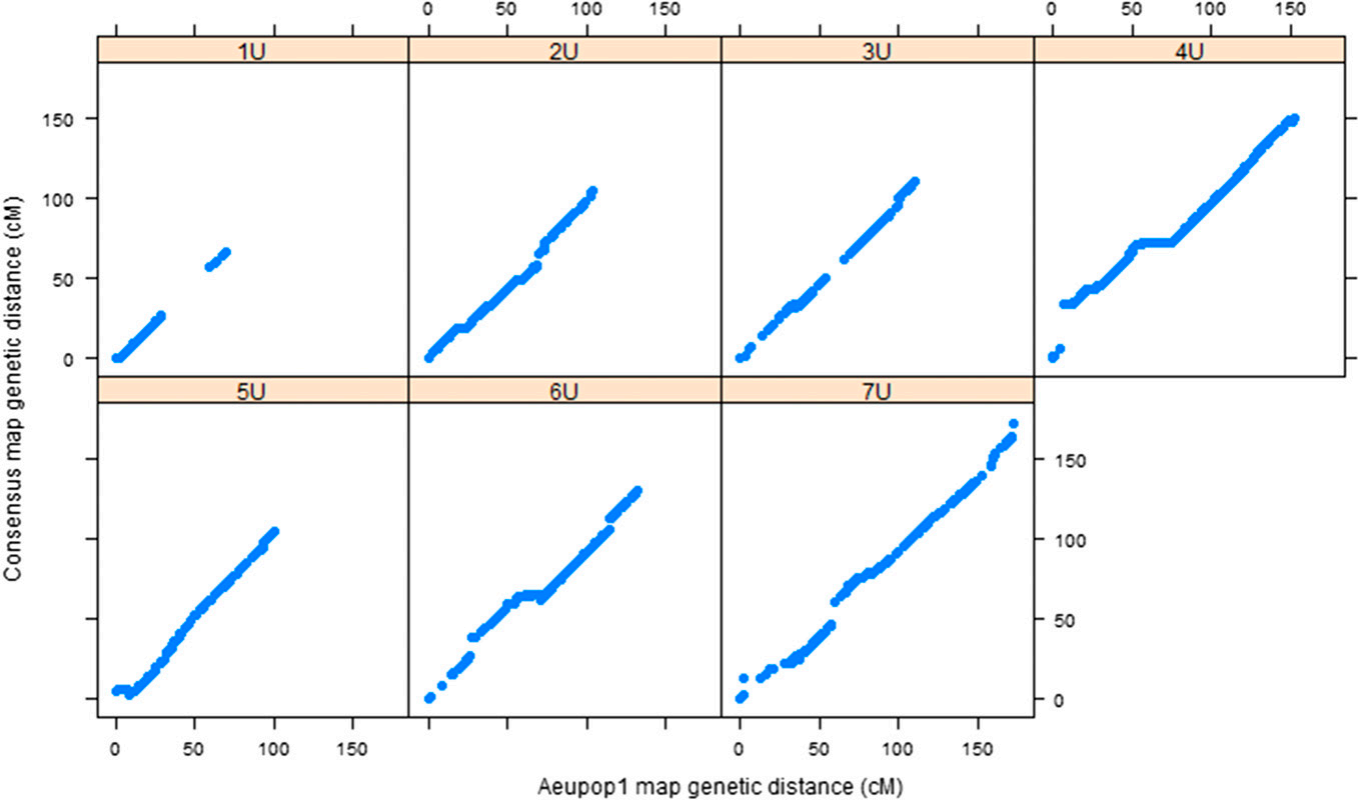
Relationship between *Aeupop1* map and consensus map. The *x-axis* shows the genetic distance (centimorgan) linkage map from *Aeupop1*, and the *y-axis* is the genetic distance (centimorgan) of the consensus map.

**Figure 4:**
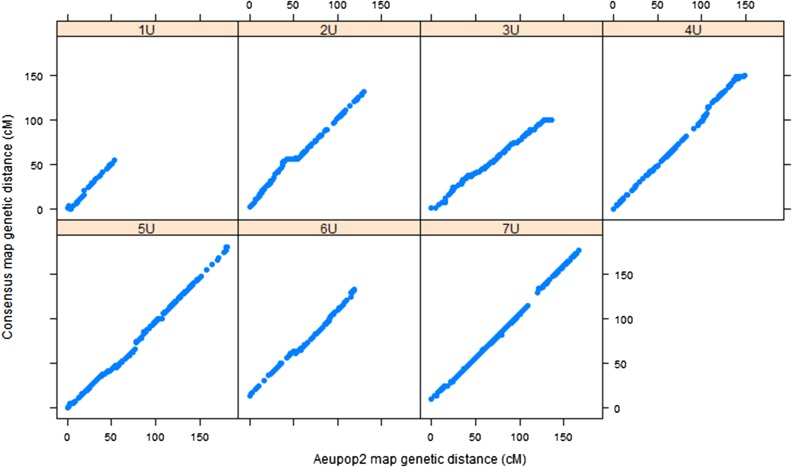
Relationship between *Aeupop2* map and consensus map. The *x-axis* shows the genetic distance (centimorgan) linkage map from *Aeupop2*, and the *y-axis* is genetic distance (centimorgan) of the consensus map.

The framework consensus linkage map’s size per chromosome was in the range of 66.6 cM (1U)–181 cM (5U), with a total map size of 948.7 cM. Chromosome 1U was consistently found as the shortest of all chromosomes across the mapping populations (Table S1). It had also the least number of markers with 1.2 SNPs/cM, whereas chromosome 7U had the largest number of markers (3.3 SNPs/cM). The relationship of the framework consensus map with the component linkage maps was assessed by comparing the genetic position of each marker on individual linkage maps with the genetic position of the framework consensus map for each population (Figure 3 and Figure 4). Spearman rank correlation (*ρ*) values of marker order between consensus map and component maps were calculated for each chromosome for both populations. The result showed that there was strong agreement between component maps and framework consensus maps in marker order for all chromosomes with *ρ* >0.999 for all chromosomes. Such very high rank correlation coefficients have also been reported for the robust framework consensus map constructed for durum wheat (Maccaferri *et al.* 2014). However, some inconsistencies in marker order were detected in each chromosome in the current study, and the corresponding markers were excluded by the algorithm implemented in the LPmerge software during consensus map construction. The probable cause for marker order conflicts may be due to biological factors such as chromosomal segment rearrangements, segmental duplications, and differences in recombination frequencies caused by genomic structural variations between the two mapping populations, as observed in different consensus map construction projects for crop species such as apple (Khan *et al.* 2012), sorghum (Mace *et al.* 2009), and durum wheat (Maccaferri *et al.* 2014). Among the nonbiological factors, missing data and genotypic errors may have contributed to the marker order conflicts observed in the current work.

### High-density consensus map construction and segregation distortion analysis

Although the framework consensus map was constructed with quality-filtered data [missing data points ≤10%, weak segregation distortion (*α* > 0.01) and no missing values for the parents], the number of SNPs placed on the framework consensus map (3009 SNPs) represented only 32.67% of the total 9210 SNPs markers that had ≤40% missing data points and were polymorphic in the *Aeupop1* or/and *Aeupop2* populations. The framework consensus map had 209 SNPs shared between the two populations, whereas the high-density consensus map was constructed based on 506 SNPs that were common between the two mapping populations. Thus, to leverage the resolution of the consensus map, we were able to place an additional 2395 markers on the component maps of the two populations using the recombination frequency approach (see *Materials and Methods* section), and the final high-density consensus map comprised a total of 5404 (58.68%) SNPs (Table S2). Moreover, these markers added to the linkage maps based on the recombination frequency method enabled us to assess the pattern of segregation distortion for each chromosome of *Ae. umbellulata*, and also to determine the syntenic relationship between *Ae. umbellulata* and other species.

**Figure 5:**
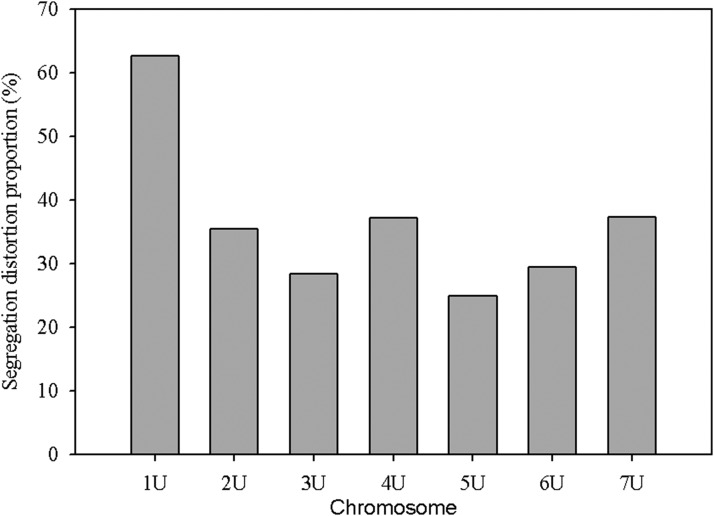
Proportion of significantly distorted markers (p ≤ 0.01) of SNP markers placed on the high density consensus map for each chromosome.

**Figure 6:**
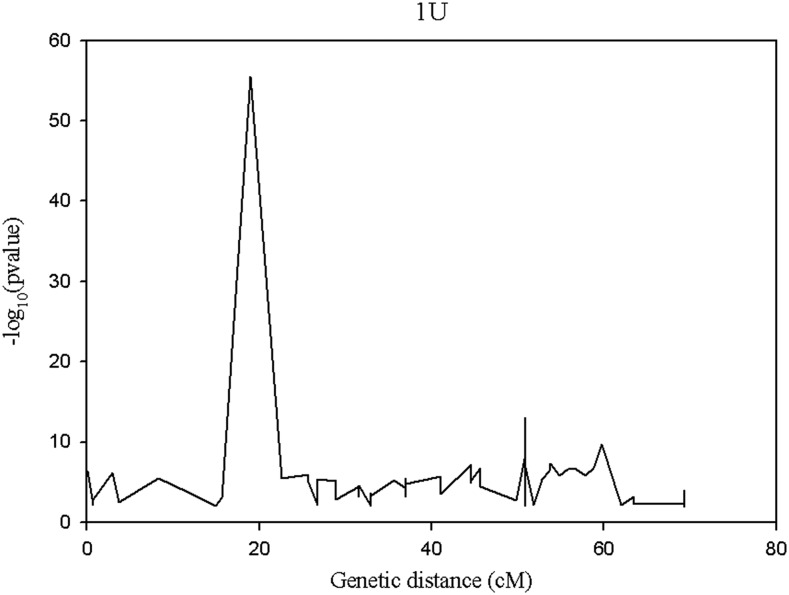
Pattern of segregation distortion across regions of chromosome 1 U. The *y-axis* indicates the segregation distortion p-value (in its negative logarithm form) for the corresponding genetic position on the *x-axis*.

**Figure 7:**
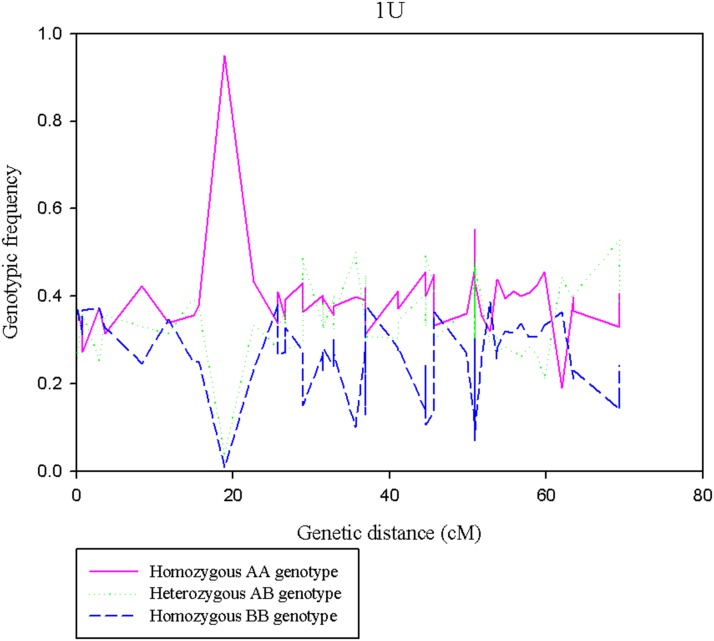
Pattern of genotypic frequencies across regions of chromosome 1U. The *y-axis* indicates the genotypic frequencies of the three genotypic classes (AA, AB, and BB) for the corresponding genetic position on the *x-axis*.

The segregation distortion analysis indicated that the proportion of distorted (*α* ≤0.01) markers was high for chromosome 1U (Figure 5), and the extremely distorted markers were located on the proximal end of the chromosome (Figure 6). Generally, over 60% of the SNPs placed on the high density map via recombination frequency values were significantly distorted for chromosome 1U, indicating segregation distortion was the major factor for the elimination of several SNP markers during filtering steps for linkage map construction for this chromosome. The remaining six chromosomes had <50% distorted markers. The patterns of segregation distortion for all chromosomes are reported in Figure S1, Figure S2, Figure S3, Figure S4, Figure S5, and Figure S6. With regard to regions with highly distorted markers, with the exception of chromosomes 1U and 7U, which each had a single highly distorted region on the short arm, all other chromosomes possessed two to five distorted regions. The nonrandom distributions of segregation distortion along chromosomal regions was previously reported for hexaploid wheat (Li *et al.* 2015), tetraploid wheat (Kumar *et al.* 2007), barley (Li *et al.* 2010), *Aegilops tauschii* (Faris *et al.* 1998), and oak (Bodenes *et al.* 2016), as well as many other plant species. Segregation distortion can occur either due to nonbiological factors such as sampling error, missing data points, and genotypic error, or due to biological factors such as gamete competition, hybrid incompatibility, deleterious alleles (genetic load), and chromosome loss or rearrangements (Tayler and Ingvarsson 2003; Alheit *et al.* 2011; Bodenes *et al.* 2016). Although the effect of technical factors cannot be ruled out, especially for weakly distorted markers that were randomly distributed across the genome, clusters of significantly distorted markers in the previously reported distorted regions for many grass species are most likely caused by genetic factors. Chromosome 1U, with the most severe segregation distortion, is homeologous to wheat group 1 chromosomes (Zhang *et al.* 1998; Molnar *et al.* 2016) and barley chromosome 1H (Edae *et al.* 2016). Segregation distortion regions have been reported on the short arms of wheat group 1 chromosome (Li *et al.* 2015), *Ae. tauschii* 1DS (Faris *et al.* 1998), and on barley 1H chromosome (Li *et al.* 2010; Belanger *et al.* 2016). Although the current study was not designed to study mechanisms that cause segregation distortion, the analysis conducted here is rather to determine the effect of segregation distortion regions on linkage map construction. As expected, linkage groups with severe segregation distortion (*e.g.*, 1U) had fewer markers, were shorter in size, and displayed inconsistent marker distribution. We also conducted similar analysis for missing data, but we have not observed noticeable effects of missing data amount on quality of linkage maps (data not shown). As to the direction of segregation distortion, in most cases, the frequency of heterozygous genotypes was either lower than the frequency of homozygous genotypes, or between the two parental frequencies (Figure 7). This could be partly due to the presence of heterozygous undercalling due to absence of deeper sequence coverage to sample both alleles in heterozygous individuals in the data sets of both mapping populations (Figure S7, Figure S8, Figure S9, Figure S10, Figure S11, and Figure S12). Overall, although it is difficult to distinguish the type of biological factors involved in segregation distortion from this study, the analysis elucidated that segregation distortion is one of the factors that adversely affected the linkage map for chromosome 1U, which was found to be the shortest chromosome with the least number of markers.

### Chromosome collinearity and synteny across Ae. umbellulata, wheat, and barley

**Figure 8:**
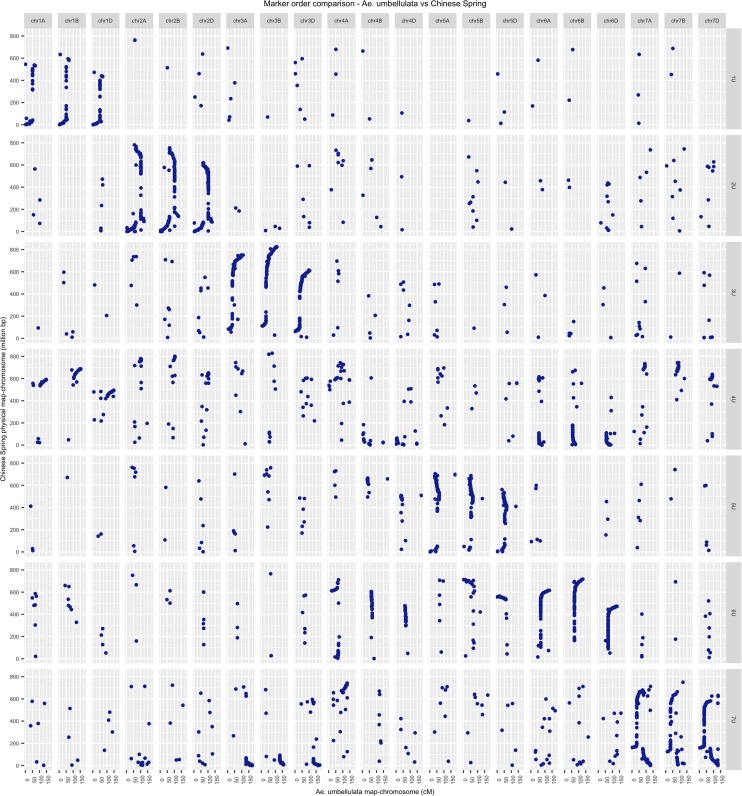
Syntenic relationship between the homeologous chromosomes of *Ae. umbellulata* and hexaploid wheat. The left *y-axis* shows Chinese Spring wheat physical map (in million base pair), and the bottom *x*-axis identifies genetic distance (centimorgan) of *Ae. umbellulata* chromosomes. The upper *x-axis* represents chromosomes of Chinese Spring wheat, whereas the left *y-axis* represents chromosomes of *Ae. umbellulata*.

The draft sequence of hexaploid wheat has greatly facilitated the assignment of linkage groups into chromosomes. The SNP tags for the markers in the high-density map (5404 markers) were aligned against the Chinese Spring assembly v0.4 (https://wheat-urgi.versailles.inra.fr/Seq-Repository/Assemblies). With this approach, we were able to assess broadly the syntenic relationship between *Ae. umbellulata* and hexaploid wheat, and also assigned all linkage groups into *Ae. umbellulata* chromosomes unambiguously using hexaploid wheat-anchored markers. A total of 3649 (67.52%) of the SNPs on the *Ae. umbellulata* high-density consensus map had unique best hits in the hexaploid genome scaffolds. Out of these SNPs, 59.69% were mapped to the expected homeologous hexaploid wheat chromosomes, *i.e.*, they showed syntentic relationships with the corresponding group of chromosomes (Figure 8). With the exception of chromosome 4U, all *Ae. umbellulata* chromosomes showed strong collinearity with corresponding homeologous chromosomes of wheat. However, chromosome 4U consisted mainly of segments of group 1, group 6, and group 7 chromosomes (Figure 8). Similarly, the SNP tags of the markers in the high-density map were integrated again into the barley genome assembly (ftp://ftpmips.helmholtz-muenchen.de/plants/barley/public_data/). A total of 802 (14.84%) SNPs had unique best hits in the barley genome assembly, and 510 (63.59%) of them were found to be syntenous. Broadly, the comparison of *Ae. umbellulata* with barley chromosomes revealed a similar syntenic pattern with that of *Ae. umbellulata* and hexaploid wheat (Figure S13). Chromosomal structural rearrangements are common in *Ae. umbellulata*, and at least 11 rearrangements have been identified with an RFLP-based genetic map between the wheat D genome and *Ae. umbellulata* chromosomes (Zhang *et al.* 1998; Devos and Gale 2000). We also detected the majority of those previously reported chromosome translocations and inversions. The major ones are translocations of short arms of 3U to 7U, the similarity of 4U with 5U, 6U, and 7U, and the chromosome inversion that spans the long arm of 2U. In addition, it appears that chromosome 6U is composed of large segments of chromosomal translocations from both 4U and 5U (Figure 8). For chromosomes 5U and 7U, we have also noticed the presence of chromosome inversions that have not been reported previously for *Ae. umbellulata*. Although we detected numerous relationships between nonhomeologous chromosomes based on single markers, we rather focused on regions that were supported by several markers as true indicators of chromosomal rearrangements as single marker-based relationship may be due to technical errors during linkage map construction and/or sequence alignment.

The use of alien segments that carry novel genes depends on the extent of chromosome collinearity and meiotic pairing of alien chromosomes with homeologous chromosomes (Devos *et al.* 1993; Lukaszewski *et al.* 2004; Devos et al. 2007; Molnar *et al.* 2016). In the current study, the level of collinearity of *Ae. umbellulata* chromosomes with homeologous chromosomes of hexaploid wheat and barley was assessed using Spearman’s rank correlation coefficients. In this line, we observed a wide range of variations among pairs of homeologous chromosomes both for hexaploid wheat and barley. Chromosomes 3U and Group 3 of hexaploid wheat (3A, 3B, and 3D) had very strong collinearity with rank correlation coefficient, *ρ* >0.9, followed by 1U and group 1 of hexaploid wheat, 6U, and group 6 of hexaploid wheat, with *ρ* values ranging from 0.6 to 0.9. Hexaploid wheat group 5 and 7 chromosomes showed low to moderated collinearity with *Ae. umbellulata* counterpart chromosomes (Figure S13). Using rank correlation, a similar pattern of collinearity was also observed between homeologous chromosomes of *Ae. umbellulata* and barley. Chromosomes 4U has no relationship with its homeologous chromosomes of hexaploid wheat as it shared multiple segments with other nonhomeologous chromosomes such as group 1, 6, and 7 of hexaploid wheat, and this was confirmed again with its chromosome collinearity with 1H, 6H, and 7H of barley (Figure 8 and Figure S13). The current results also agree with our previous analysis based on the data from a single population (Edae *et al.* 2016).

### GBS SNP annotation with gene models and transcripts

Similarity search of GBS SNP tags in gene models and cDNA libraries of other cereal crops revealed that ∼6% of the *Ae. umbellulata* SNPs were in genic regions (Table 2). However, through integration of GBS tags into transcript assembly of *Ae. umbellulata* itself indicated that ∼20% of GBS SNPs are genic SNPs. GBS genic SNP estimation has also been estimated for oat (5%) (Huang *et al.* 2014) and barley (10.2%) (Kono *et al.* 2013). In the current work, we found similar results as to the integration of GBS SNPs into other species, but the higher genic GBS SNPs from *Ae. umbellulata* transcripts is likely because tissue sources for the transcript assemblies of the latter were from two parents of the four parents of the mapping populations used in this study. Nonetheless, the presence of GBS SNPs in the expressed portion of a genome implies that there is a possibility to identify functional SNPs, especially with high density SNPs, through QTL mapping and association mapping approaches using GBS technology.

**Table 2:** Similarity of *Ae. umbellulata* SNP tags with gene models and cDNA transcripts of *Ae. umbellulata*, hexaploid wheat, barley, *Ae. tauschii*, and *T. urartu*

Species	No. of Best Matches	Type of Database	% Best Matches
*Ae. umbellulata*	8091	RNA-seq assembly	19.29
*Ae. umbellulata*	8737	RNA-seq assembly	20.84
Hexaploid wheat	2549	Gene model	6.08
Barley	1312	Gene model	3.13
*Ae. tauschii*	2659	cDNA library	6.34
*T. urartu*	2373	cDNA library	5.66

The use of two mapping populations allowed us to place a high number of GBS SNPs on a single consensus map. To date this is the only high-density consensus map constructed for *Ae. umbellulata*. The consensus map is developed with the belief that its availability will greatly facilitate genomic/genetics studies including the identification of novel QTL, refining QTL positions, gene cloning and genome assembly in *Ae. umbellulata*. Specifically, the mapping of novel stem rust resistance from this species has already started with the first population (*Aeupop1*) and a major QTL for two stem rust races has been detected on chromosome 2U (Edae *et al.* 2016). Therefore, mapping resistance QTL for the same races will be done using the second population (*Aeupop2*). We hope that this consensus map will help to confirm and refine the genetic position of the mapped QTL as the use of GBS-based linkage maps for precise QTL mapping and facilitation of gene cloning for different traits has been shown in several previous studies (Kujur *et al.* 2016; Pootakham *et al.* 2015; Balsalobre *et al.* 2017).

## Supplementary Material

Supplemental material is available online at www.g3journal.org/lookup/suppl/doi:10.1534/g3.117.039966/-/DC1.
