# Supplementary figures and images for: Genotyping-by-Sequencing Facilitates a High-Density Consensus Linkage Map for Aegilops umbellulata, a Wild Relative of Cultivated Wheat
Source: G3 (Bethesda). 2017 Mar 29;7(5):1551–61. doi: 10.1534/g3.117.039966 (PMC5427507; doi:10.1534/g3.117.039966)

2U

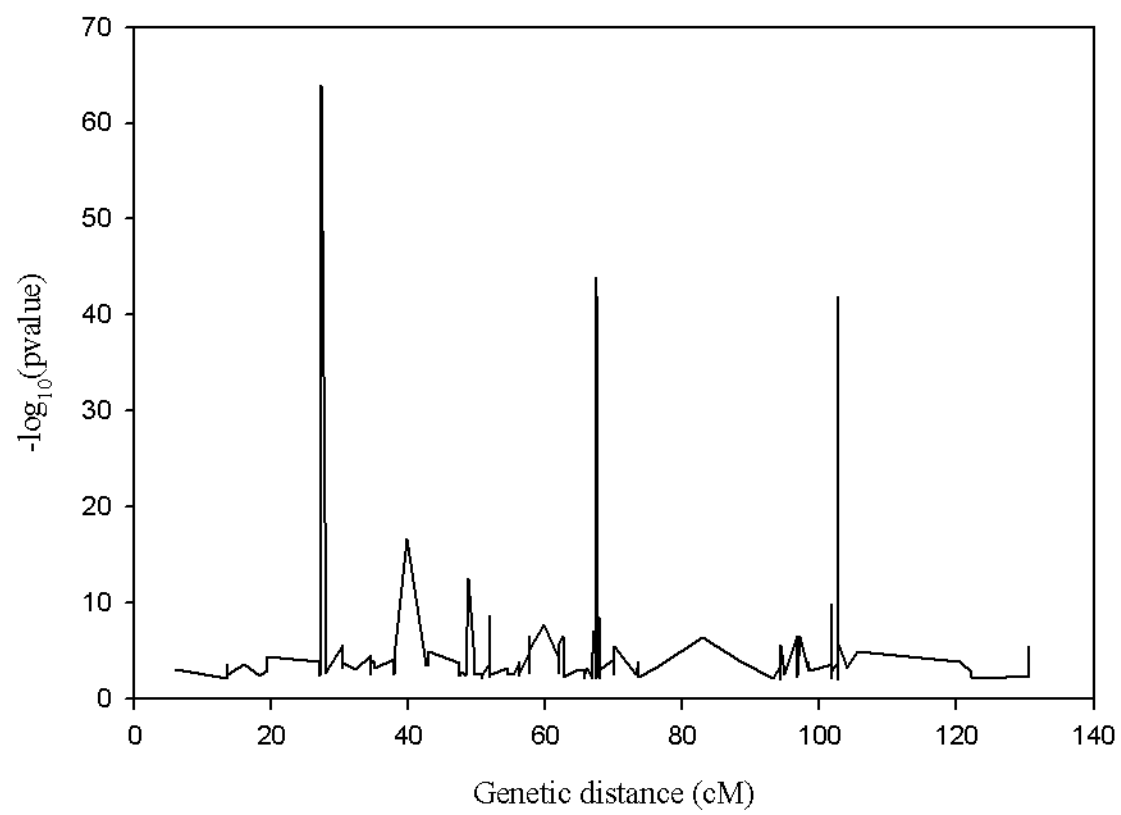

Supplement: Supplementary file 1 [file 1551FigureS1.pdf]

3U

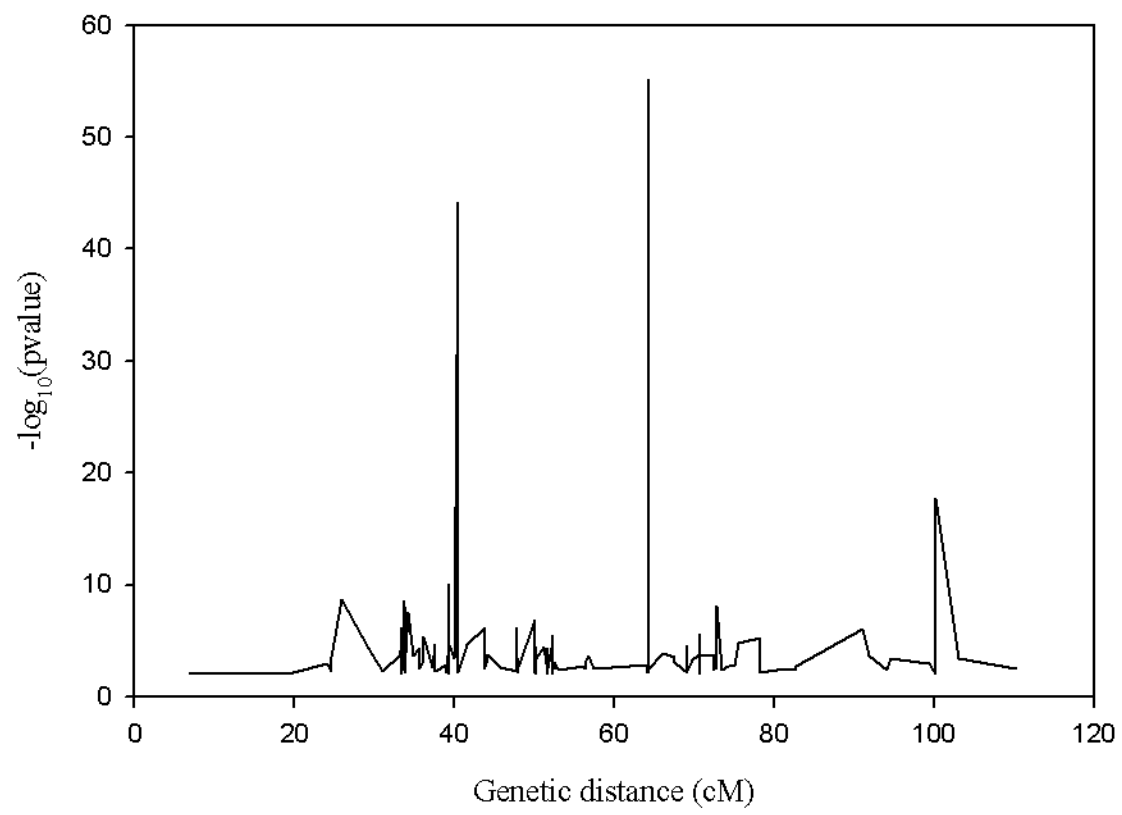

Supplement: Supplementary file 2 [file 1551FigureS2.pdf]

4U

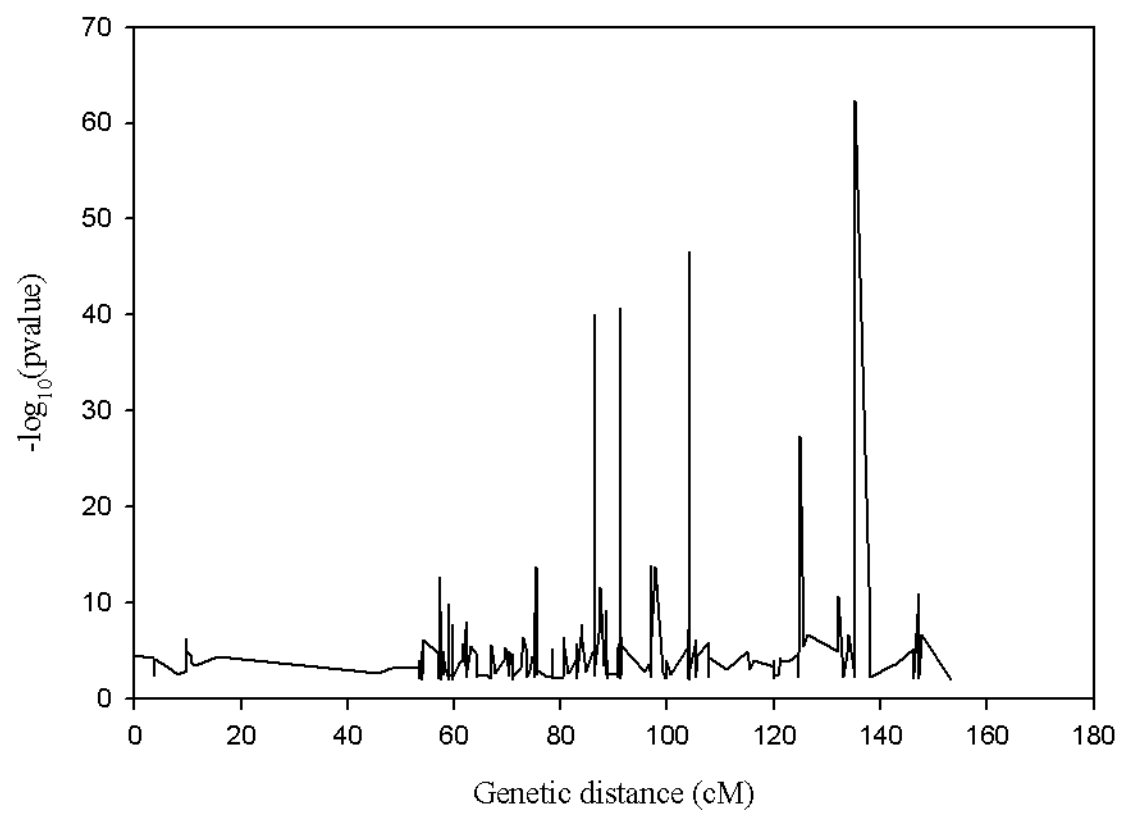

Supplement: Supplementary file 3 [file 1551FigureS3.pdf]

5U

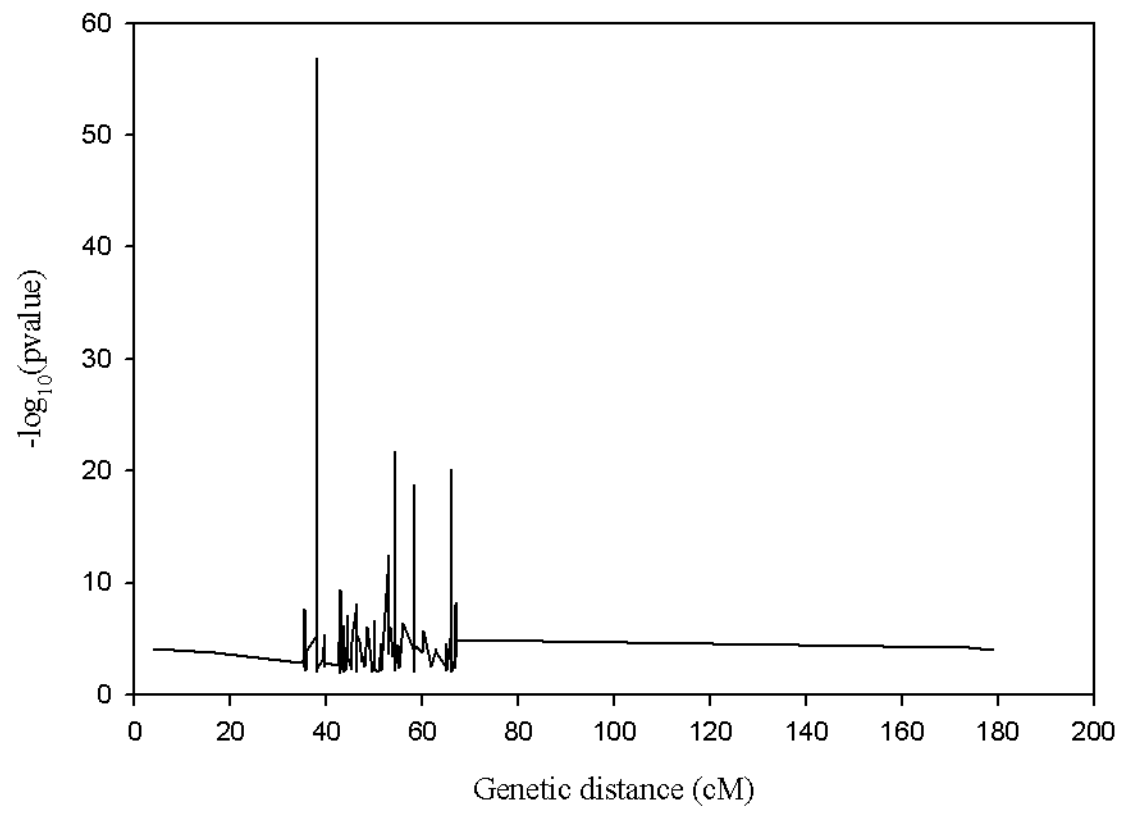

Supplement: Supplementary file 4 [file 1551FigureS4.pdf]

6U

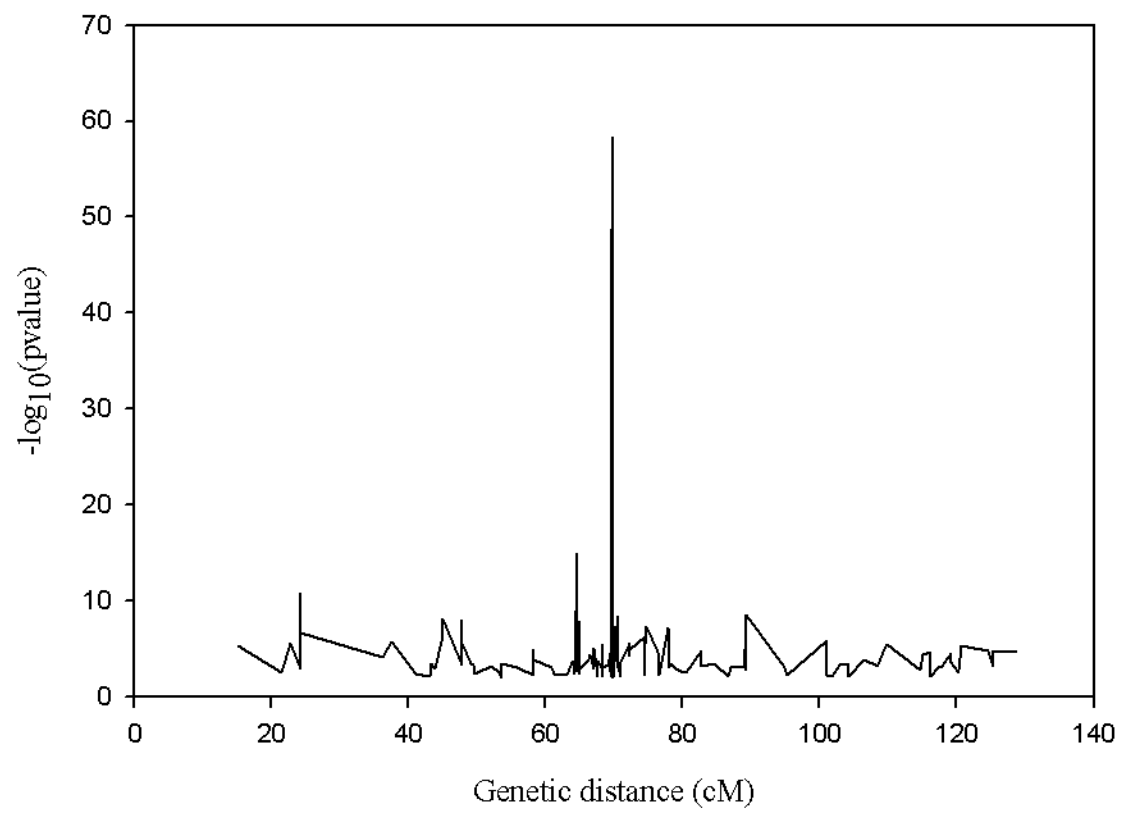

Supplement: Supplementary file 5 [file 1551FigureS5.pdf]

7U

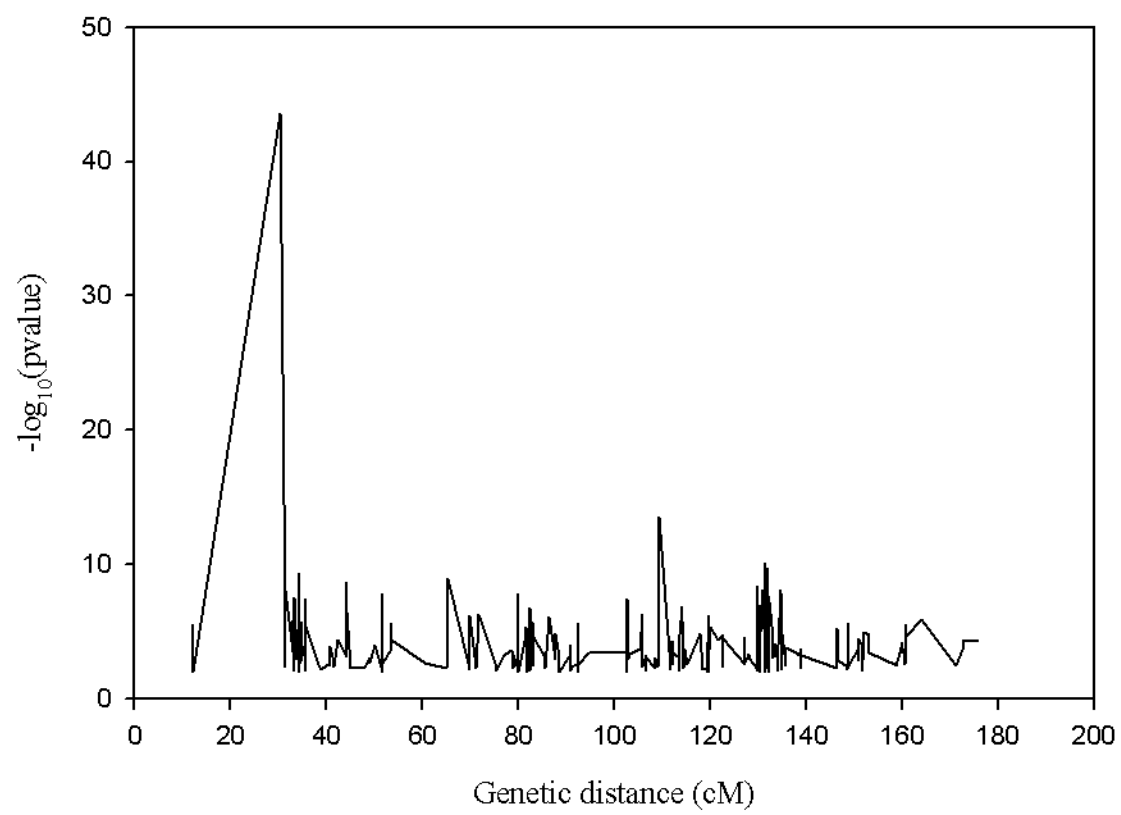

Supplement: Supplementary file 6 [file 1551FigureS6.pdf]

2U

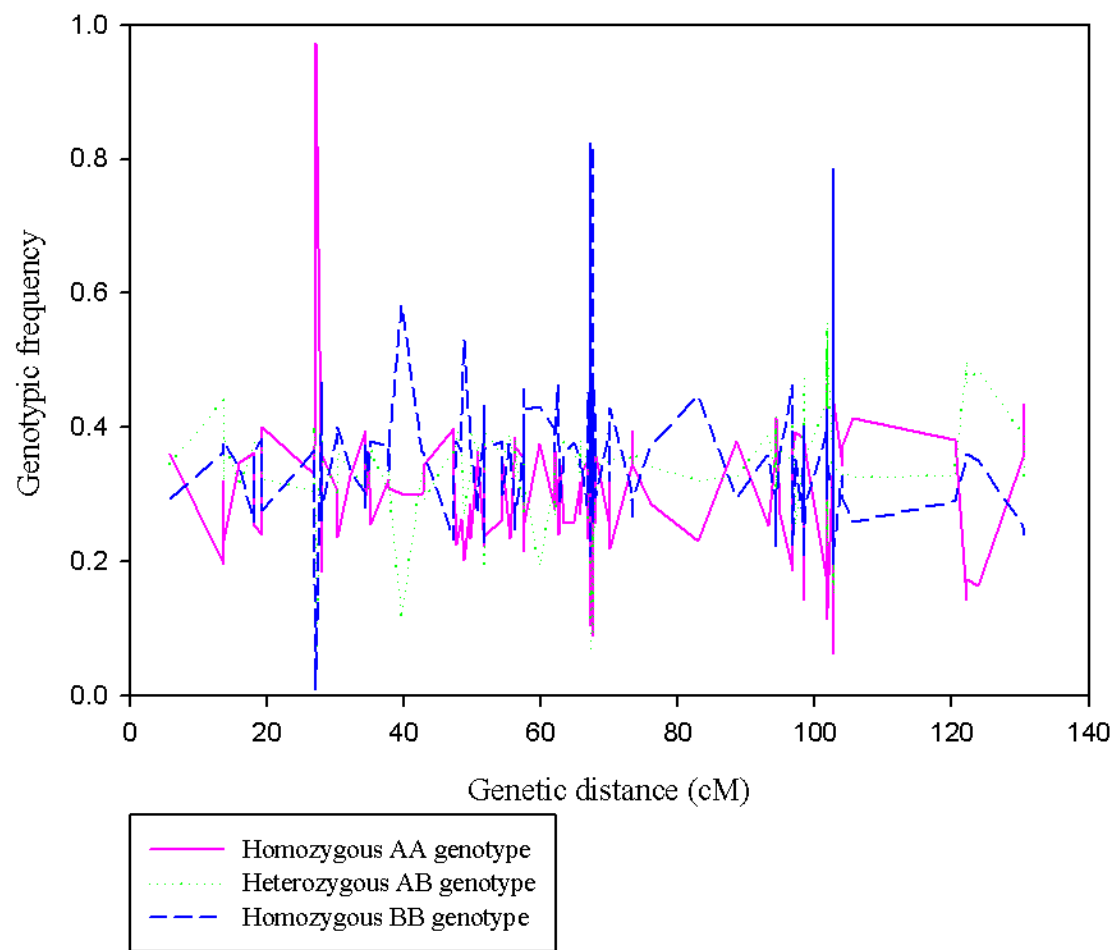

Supplement: Supplementary file 7 [file 1551FigureS7.pdf]

3U

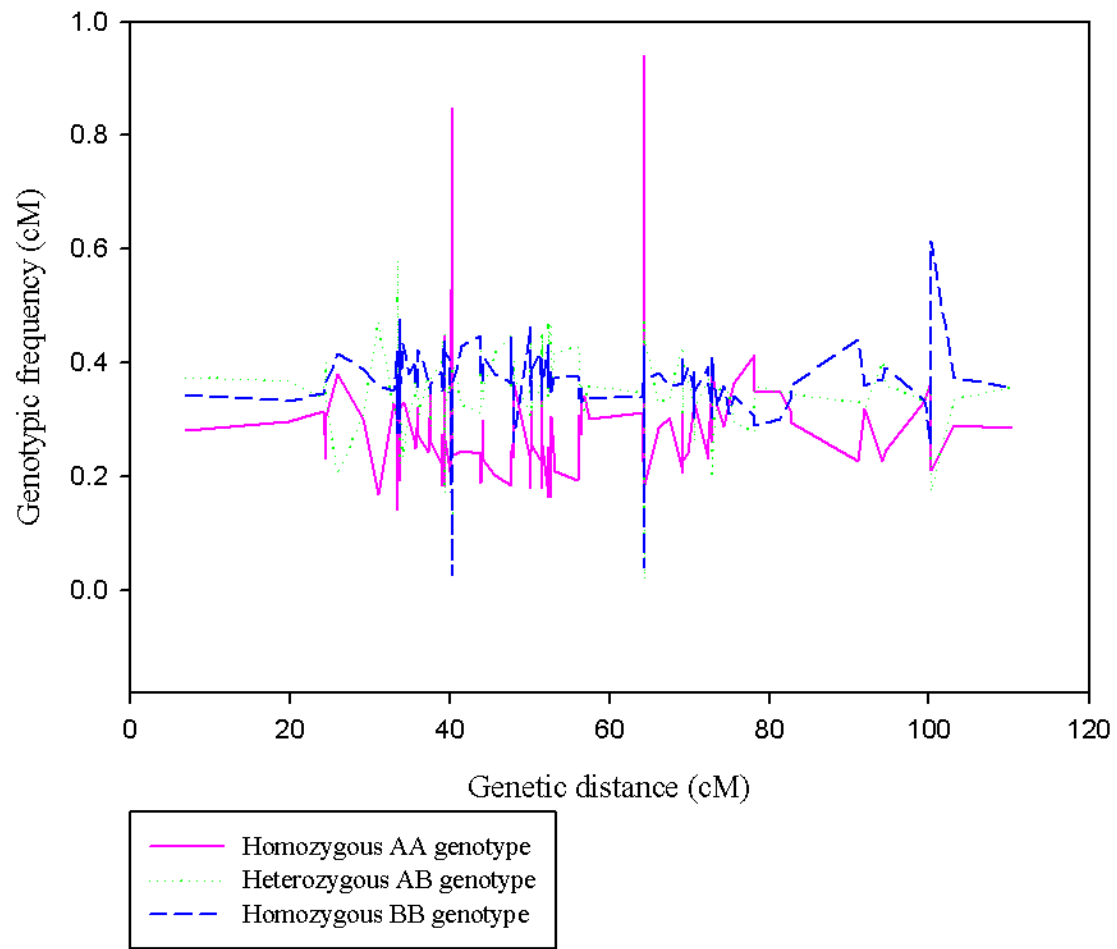

Supplement: Supplementary file 8 [file 1551FigureS8.pdf]

4U

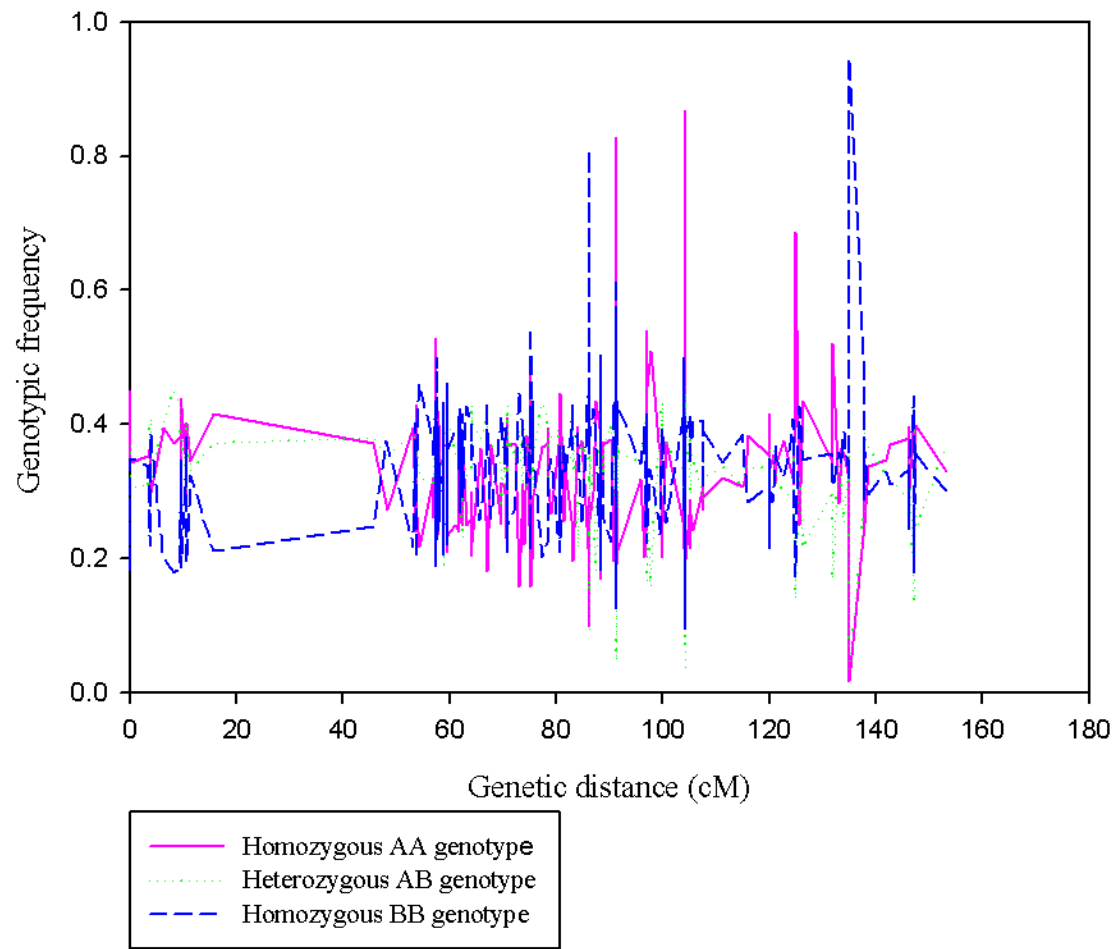

Supplement: Supplementary file 9 [file 1551FigureS9.pdf]

5U

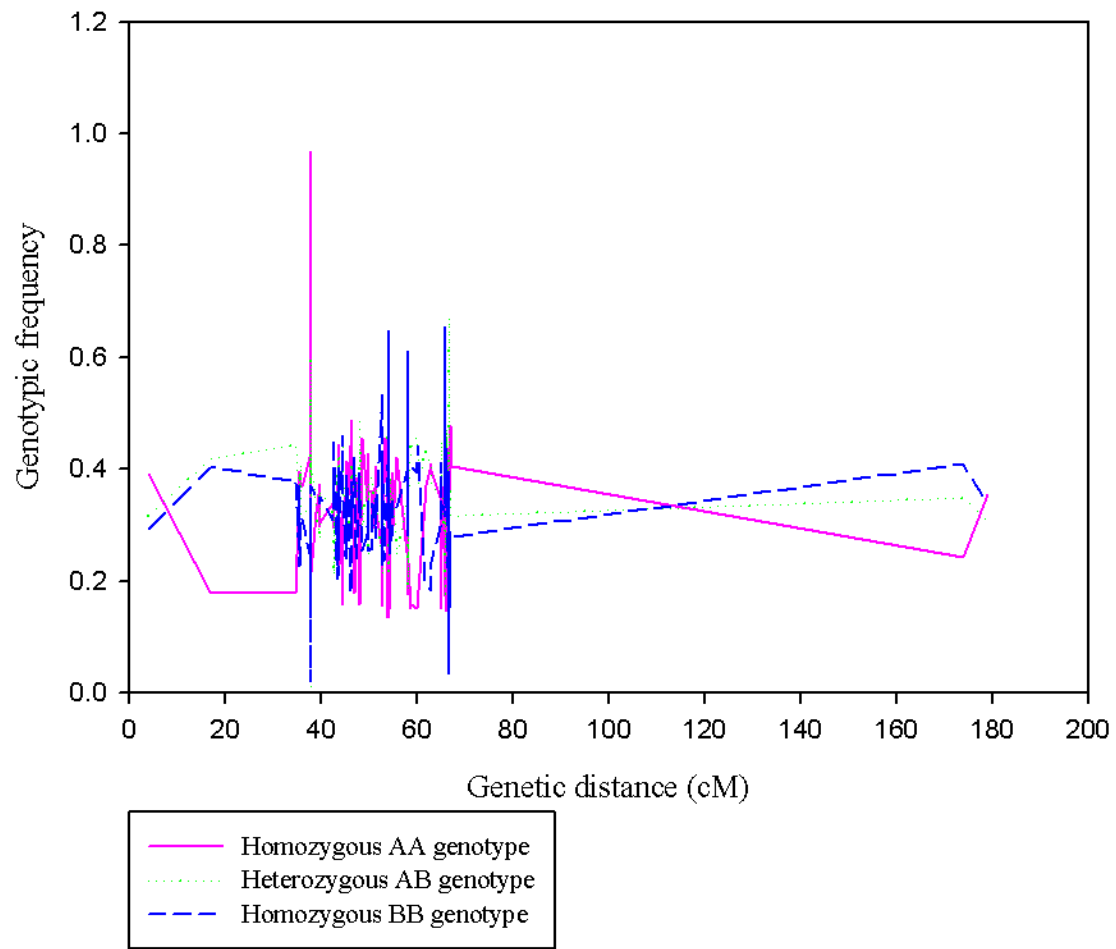

Supplement: Supplementary file 10 [file 1551FigureS10.pdf]

6U

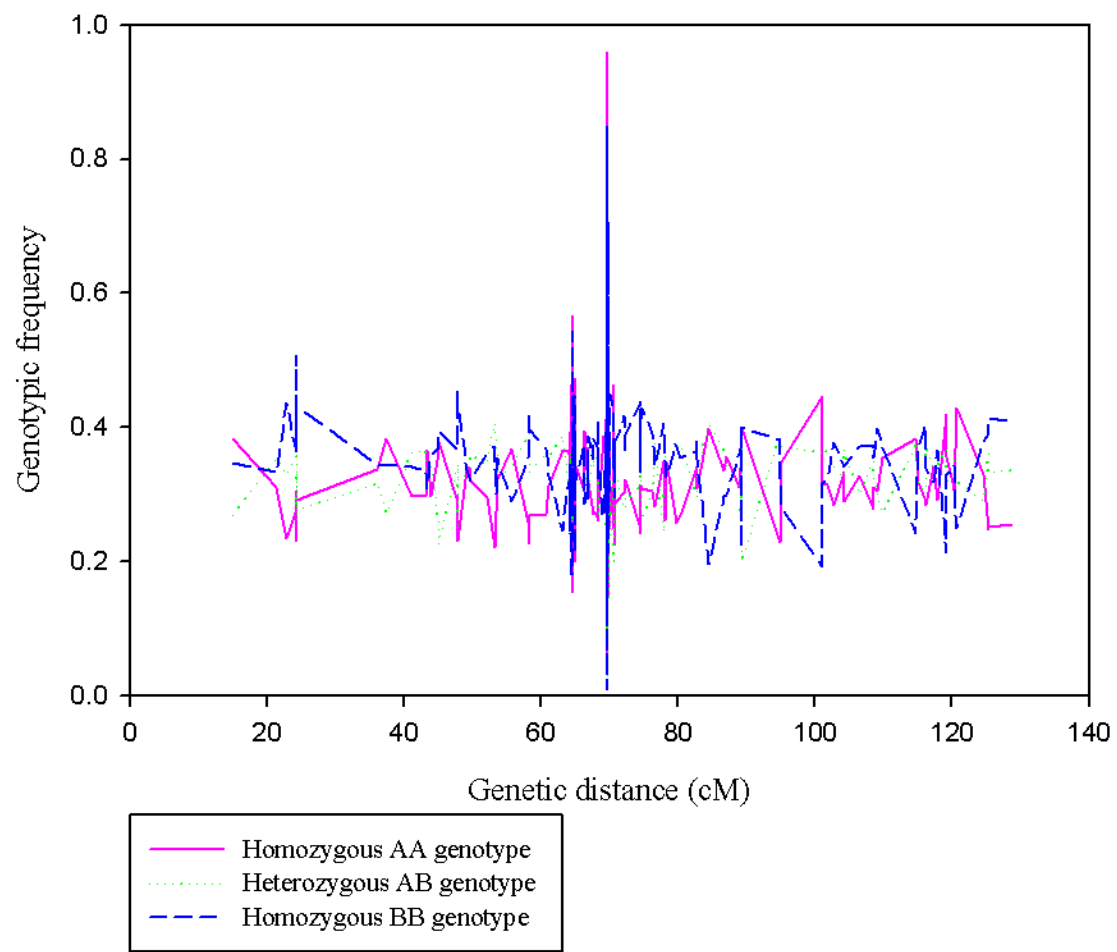

Supplement: Supplementary file 11 [file 1551FigureS11.pdf]

7U

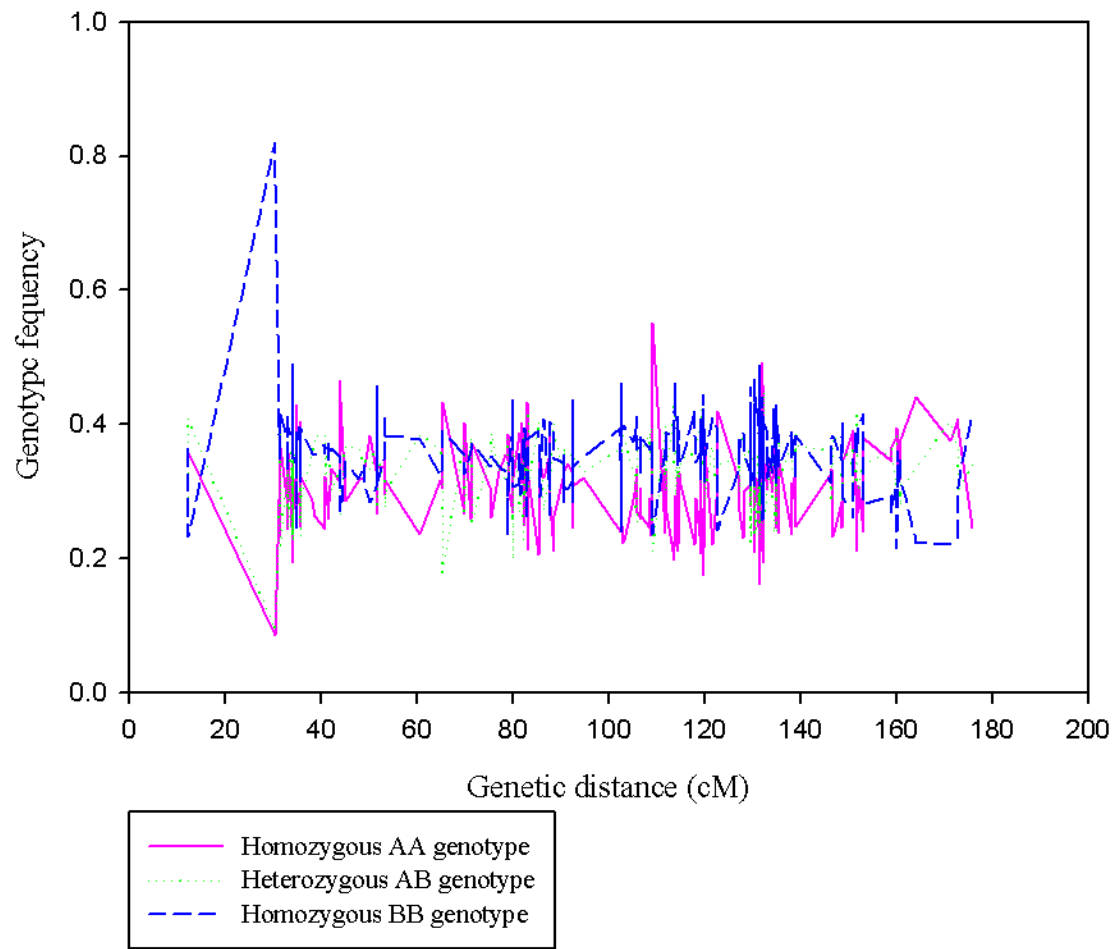

Supplement: Supplementary file 12 [file 1551FigureS12.pdf]

Marker order comparison - Ae. umbellulata vs barley

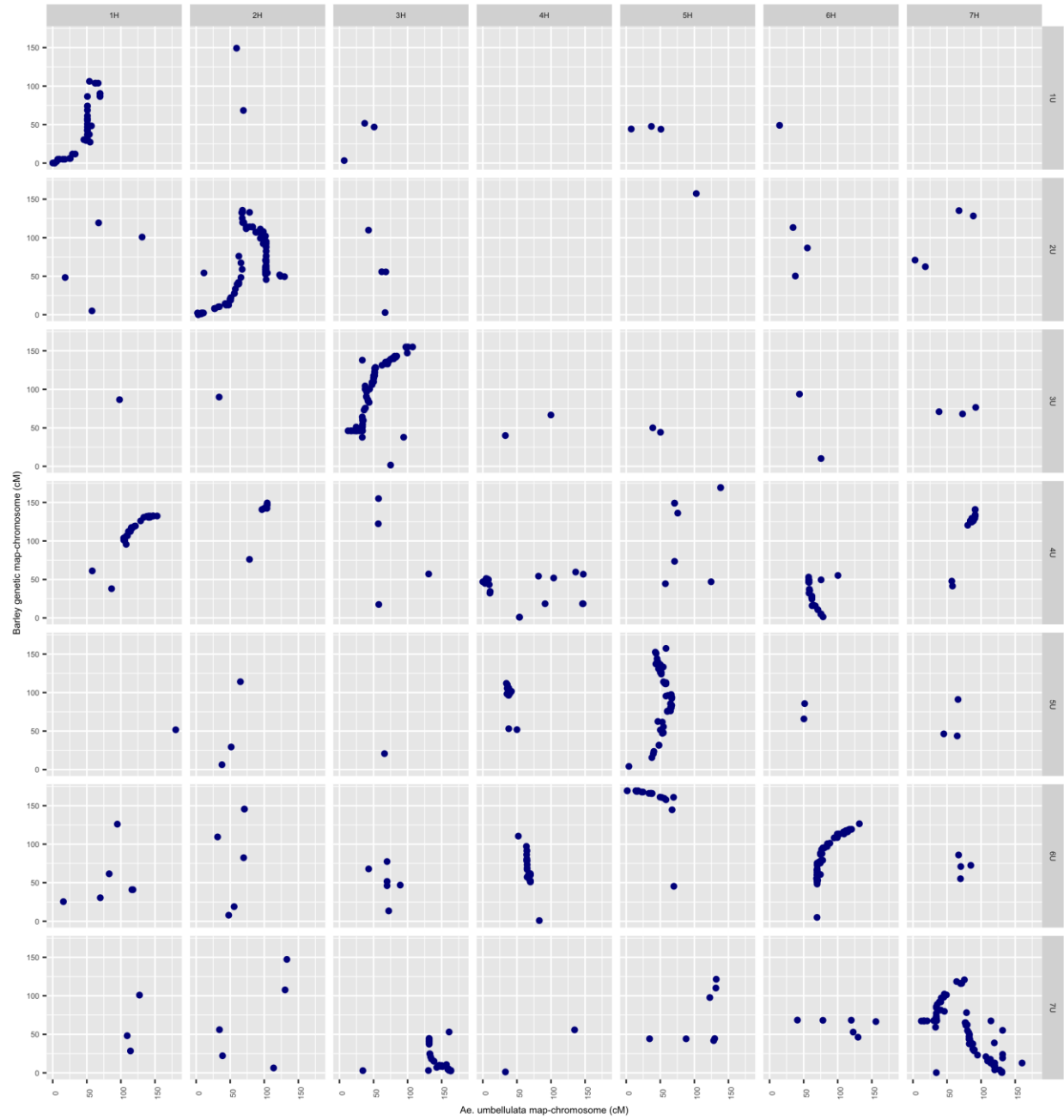

Supplement: Supplementary file 13 [file 1551FigureS13.pdf]
