# Supplementary material for: Genotyping-by-Sequencing Facilitates a High-Density Consensus Linkage Map for Aegilops umbellulata, a Wild Relative of Cultivated Wheat
Source: G3 (Bethesda). 2017 Mar 29;7(5):1551–61. doi: 10.1534/g3.117.039966 (PMC5427507; doi:10.1534/g3.117.039966)
Supplement: Supplementary file 16 [file 1551TableS3.docx]

Table S3. Spearman’s rank correlation coefficients of marker order on chromosomes of *Ae. umbellulata* in relation to order on hexaploid wheat and barley chromosomes.

| Species | Chromosomes | Correlation coefficient (*rho*) | Number of markers | p-value |
| --- | --- | --- | --- | --- |
| *Ae. umbellulata* vs hexaploid wheat | 1U vs 1A | 0.783 | 45 | 7.36e-11 |
|  | 1U vs 1B | 0.670 | 41 | 1.68e-06 |
|  | 1U vs 1D | 0.732 | 57 | 9.45e-11 |
|  | 2U vs 2A | 0.572 | 181 | 2.2e-16 |
|  | 2U vs 2B | 0.463 | 126 | 4.765e-08 |
|  | 2U vs 2D | 0.433 | 198 | 1.85e-10 |
|  | 3U vs 3A | 0.952 | 117 | 2.2e-16 |
|  | 3U vs 3B | 0.942 | 99 | 2.2e-16 |
|  | 3U vs 3D | 0.952 | 46 | 2.2e-16 |
|  | 4U vs 4A | 0.103 | 26 | 0.616 |
|  | 4U vs 4B | -0.515 | 21 | 0.017 |
|  | 4U vs 4D | -0.005 | 33 | 0.977 |
|  | 5U vs 5A | -0.270 | 110 | 0.0043 |
|  | 5U vs 5B | -0.362 | 91 | 0.00042 |
|  | 5U vs 5D | -0.279 | 121 | 0.0020 |
|  | 6U vs 6A | 0.900 | 120 | 2.2e-16 |
|  | 6U vs 6B | 0.776 | 89 | 2.2e-16 |
|  | 6U vs 6D | 0.790 | 146 | 2.2e-16 |
|  | 7U vs 7A | -0.719 | 152 | 2.2e-16 |
|  | 7U vs 7B | -0.226 | 81 | 0.0425 |
|  | 7U vs 7D | -0.540 | 177 | 9.017e-15 |
| *Ae. umbellulata* vs barley | 1U vs 1H | 0.899 | 45 | 2.2e-16 |
|  | 2U vs 2H | 0.416 | 117 | 3.103e-06 |
|  | 3U vs 3H | 0.866 | 94 | 2.2e-16 |
|  | 4U vs 4H | -0.008 | 18 | 0.976 |
|  | 5U vs 5H | -0.157 | 59 | 0.233 |
|  | 6U vs 6H | 0.888 | 83 | 2.2e-16 |
|  | 7U vs 7H | -0.751 | 94 | 2.2e-16 |
